# Methane on Mars and Habitability: Challenges and Responses

**DOI:** 10.1089/ast.2018.1917

**Published:** 2018-10-12

**Authors:** Yuk L. Yung, Pin Chen, Kenneth Nealson, Sushil Atreya, Patrick Beckett, Jennifer G. Blank, Bethany Ehlmann, John Eiler, Giuseppe Etiope, James G. Ferry, Francois Forget, Peter Gao, Renyu Hu, Armin Kleinböhl, Ronald Klusman, Franck Lefèvre, Charles Miller, Michael Mischna, Michael Mumma, Sally Newman, Dorothy Oehler, Mitchio Okumura, Ronald Oremland, Victoria Orphan, Radu Popa, Michael Russell, Linhan Shen, Barbara Sherwood Lollar, Robert Staehle, Vlada Stamenković, Daniel Stolper, Alexis Templeton, Ann C. Vandaele, Sébastien Viscardy, Christopher R. Webster, Paul O. Wennberg, Michael L. Wong, John Worden

**Affiliations:** ^1^ California Institute of Technology, Pasadena, California.; ^2^ NASA Jet Propulsion Laboratory, California Institute of Technology, Pasadena, California.; ^3^ University of Southern California, Los Angeles, California.; ^4^ University of Michigan, Ann Arbor, Michigan.; ^5^ University of California, Davis, California.; ^6^ NASA Ames Research Center, Blue Marble Space Institute of Science, Mountain View, California.; ^7^ Istituto Nazionale di Geofisica e Vulcanologia, Rome, Italy.; ^8^ Faculty of Environmental Science and Engineering, Babes-Bolyai University, Cluj-Napoca, Romania.; ^9^ The Pennsylvania State University, University Park, Pennsylvania.; ^10^ Laboratoire de Météorologie Dynamique, Institut Pierre Simon Laplace, CNRS, Paris, France.; ^11^ University of California, Berkeley, California.; ^12^ Colorado School of Mines, Golden, Colorado.; ^13^ Laboratoire Atmospheres, Milieux, Observations Spatiales (LATMOS), IPSL, Paris, France.; ^14^ NASA Goddard Space Flight Center, Greenbelt, Maryland.; ^15^ Planetary Science Institute, Tucson, Arizona.; ^16^ US Geological Survey, Menlo Park, California.; ^17^ University of Toronto, Toronto Ontario, Canada.; ^18^ University of Colorado, Boulder, Colorado.; ^19^ The Royal Belgian Institute for Space Aeronomy (BIRA-IASB), Brussels, Belgium.

**Keywords:** Mars, CH_4_, Subsurface redox conditions, Mars instrumentation

## Abstract

Recent measurements of methane (CH_4_) by the Mars Science Laboratory (MSL) now confront us with robust data that demand interpretation. Thus far, the MSL data have revealed a baseline level of CH_4_ (∼0.4 parts per billion by volume [ppbv]), with seasonal variations, as well as greatly enhanced spikes of CH_4_ with peak abundances of ∼7 ppbv. What do these CH_4_ revelations with drastically different abundances and temporal signatures represent in terms of interior geochemical processes, or is martian CH_4_ a biosignature? Discerning how CH_4_ generation occurs on Mars may shed light on the potential habitability of Mars. There is no evidence of life on the surface of Mars today, but microbes might reside beneath the surface. In this case, the carbon flux represented by CH_4_ would serve as a link between a putative subterranean biosphere on Mars and what we can measure above the surface. Alternatively, CH_4_ records modern geochemical activity. Here we ask the fundamental question: how active is Mars, geochemically and/or biologically? In this article, we examine geological, geochemical, and biogeochemical processes related to our overarching question. The martian atmosphere and surface are an overwhelmingly oxidizing environment, and life requires pairing of electron donors and electron acceptors, that is, redox gradients, as an essential source of energy. Therefore, a fundamental and critical question regarding the possibility of life on Mars is, “Where can we find redox gradients as energy sources for life on Mars?” Hence, regardless of the pathway that generates CH_4_ on Mars, the presence of CH_4_, a reduced species in an oxidant-rich environment, suggests the possibility of redox gradients supporting life and habitability on Mars. Recent missions such as ExoMars Trace Gas Orbiter may provide mapping of the global distribution of CH_4_. To discriminate between abiotic and biotic sources of CH_4_ on Mars, future studies should use a series of diagnostic geochemical analyses, preferably performed below the ground or at the ground/atmosphere interface, including measurements of CH_4_ isotopes, methane/ethane ratios, H_2_ gas concentration, and species such as acetic acid. Advances in the fields of Mars exploration and instrumentation will be driven, augmented, and supported by an improved understanding of atmospheric chemistry and dynamics, deep subsurface biogeochemistry, astrobiology, planetary geology, and geophysics. Future Mars exploration programs will have to expand the integration of complementary areas of expertise to generate synergistic and innovative ideas to realize breakthroughs in advancing our understanding of the potential of life and habitable conditions having existed on Mars. In this spirit, we conducted a set of interdisciplinary workshops. From this series has emerged a vision of technological, theoretical, and methodological innovations to explore the martian subsurface and to enhance spatial tracking of key volatiles, such as CH_4_.

## 1. Introduction

A potential biosignature is a feature that is consistent with biological processes and that, when it is encountered, challenges the researcher to attribute it either to inanimate or to biological processes. Such detection might compel investigators to gather more data before reaching a conclusion as to the presence or absence of life. (NASA Astrobiology Roadmap, 2008, p. 15)

The scientific significance of any potential sign of past life comes not only from the probability of life having produced it, but also from the improbability of non-biological processes producing it. (Mars 2020 Science Definition Team report)

Methane (CH_4_) is a potential biosignature on Mars. Recent measurements of CH_4_ by the Mars Science Laboratory (MSL) have challenged the scientific community to explain the seemingly unexplainable, according to known chemical and physical processes on Mars (see Lefèvre and Forget, 2009; Yung and Chen, 2015; and discussions below). The possibility of a Mars that is biologically or geochemically active now confronts us more firmly than ever. Rising to the challenge, a group of interdisciplinary researchers conducted two workshop studies under the auspices of the Keck Institute for Space Studies. We aimed to synthesize innovative concepts for future Mars investigations focused on ascertaining the origin of methane on Mars. This article reports our recommendations in this regard. Mars offers a combination of properties of unique astrobiological importance: (1) Water/rock reactions, which can provide redox energy for life on encountering oxidants, once took place in the subsurface and on the surface of Mars and might still be taking place under the surface. (2) The general history of Mars is one of degrading habitability, such that the present martian surface is arid and ubiquitously oxidizing, inhospitable to life. (3) Of the Venus/Earth/Mars trio, Mars is the only one presenting an observable ∼4 billion-year geological record of climate and water-related processes. Thus, understanding whether life ever existed on Mars can yield unique knowledge regarding the limits of habitability. Methane provides a key clue.

There are several varieties of potential past and present sources for methane on Mars. These can be abiogenic or biogenic.

Abiogenic sources include the following:

- Reduction of C by H_2_ via Fischer–Tropsch-type (FTT) reactions (*e.g.*, Sabatier reaction or CO_2_ hydrogenation), where H_2_ is previously produced during iron oxidation, serpentinization, and/or radiolysis.
- UV alteration (Keppler *et al.*, 2012) of organics delivered to the planet by meteorites.
- Volcanically degassed CH_4_ (although this source is not an important CH_4_ emitter on Earth) or ancient volcanic methane stored in clathrates (Chastain and Chevrier, 2007).
- Impact metamorphism of meteoritically delivered organics (Oehler *et al.*, 2005; Oehler and Etiope, 2017).

Biogenic sources include the following:

- CH_4_ produced by now extinct or extant methanogenic microorganisms.
- If life ever developed on Mars, then there probably are regions where remnant organic matter is preserved in the subsurface. Heating by, for example, impact metamorphism could enable conversion to CH_4_, which would then be trapped in sealed reservoirs today (Oehler *et al.*, 2005; Oehler and Etiope, 2017).

**FIG. 1.**
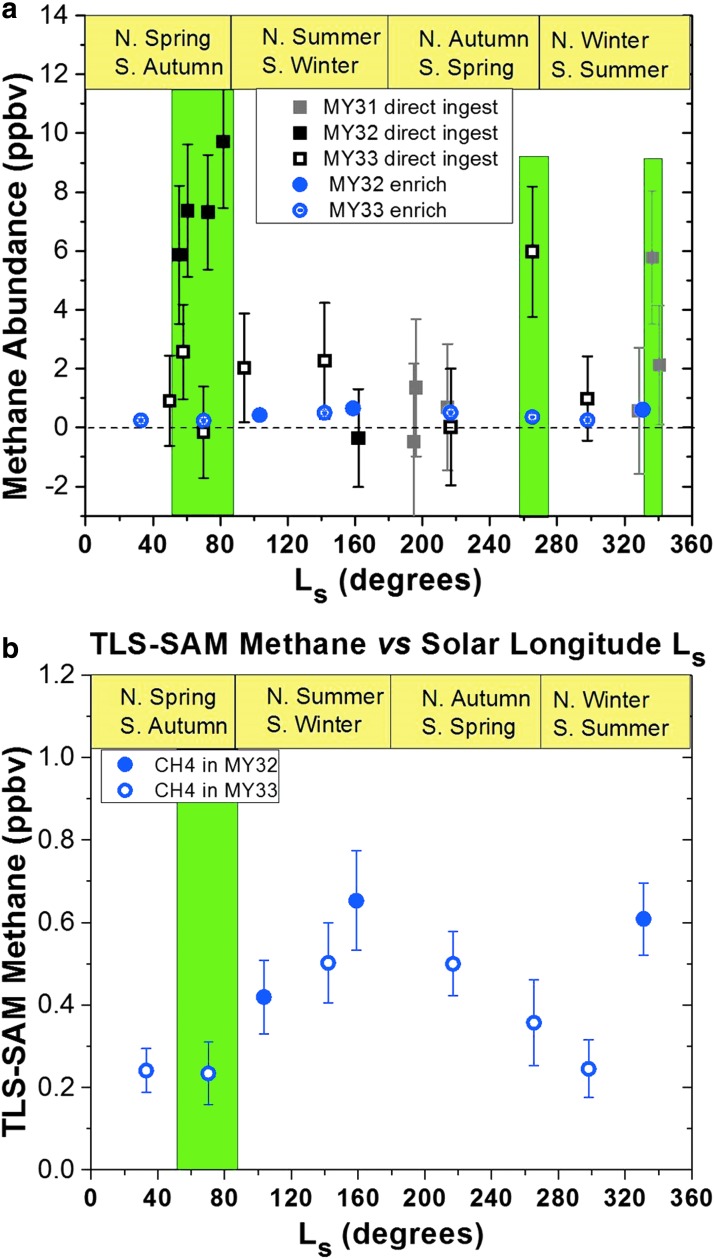
Summary of TLS measurements of CH_4_, both **(a)** including and **(b)** excluding the high spike data from year 1 (ca. January 2014). Data from Webster *et al.* (2015, 2018). TLS, Tunable Laser Spectrometer.

MSL detections of CH_4_ and the observed temporal patterns (Webster *et al.*, 2015; see Fig. 1a, b) now confront us with unambiguous data that demand interpretation. As discussed in Section 2, the MSL measurements of CH_4_ are summarized in the following observations:

(1) There is a “background” level of 0.2–0.8 parts per billion by volume (ppbv).
(2) A seasonal pattern in this low-level CH_4_ is emerging from the set of year-over-year measurements to date (Webster *et al.*, 2018).
(3) Three spikes of CH_4_ with peak values of 6–10 ppbv have been observed.

These observations pose fundamental challenges to our current understanding of Mars. First, according to known atmospheric chemistry, the gas-phase lifetime of CH_4_ is about 300 years (Summers *et al.*, 2002; Atreya *et al.*, 2007). Given this predicted photochemical lifetime, which is far longer than global atmospheric mixing timescales on Mars, a small source based on photolytically driven release of CH_4_ from interplanetary dust particles may be sufficient to explain the background observation (observation 1) (Webster *et al.*, 2015). However, the long lifetime of CH_4_ is incompatible with the observed variability (observation 2) (Lefèvre and Forget, 2009). This suggests that hitherto unknown physical and/or chemical processes are controlling the variability of atmospheric CH_4_. The spikes of CH_4_ (observation 3) suggest the possibility of deeper connections to martian geology, geophysics, geochemistry, and possibly biogeochemistry and astrobiology.

We address these questions in nine sections. Sections 2 through 5 address the fundamental question: How active is Mars geophysically and/or biologically? Section 2 discusses MSL measurements. Section 3 details the physical and chemical processes related to the lifetime of CH_4_. Section 4 addresses the possible geophysical, geochemical, and biogeochemical processes associated with CH_4_. Section 5 addresses issues related to seepage and measurement strategies. The three subsequent sections focus on the next steps in Mars CH_4_ science. ExoMars Trace Gas Orbiter (TGO) is expected to provide the next leap in our knowledge of methane on Mars, as discussed in Section 6, followed by a discussion in Section 7 of biosignatures and exploration of the martian subsurface. The subsurface is expected to play a major role in the search for biosignatures because it is the only place to find reductants on Mars (the surface is universally too oxidizing) and because of the potential for biopreservation. To take advantage of these expected and potential new measurements, it is essential that we develop appropriate modeling capabilities, especially related to the transport of trace species, and innovative technologies; this aspect is discussed in Section 8. Finally, Section 9 discusses our conclusions and a vision for the future exploration of Mars.

## 2. MSL Measurements

In this article, we focus on the MSL Tunable Laser Spectrometer (TLS) measurements. Previous measurements are summarized in Appendix 1. These prior remote-sensing detection claims have been questioned due to interference from telluric absorption in the ground-based observations, low spectral resolution in the orbital observations, and contradictions between the locations of maxima reported in ground-based observations compared to maps created from spacecraft data. A way to move forward is suggested in Appendix 1.

MSL's measurements indicate a background CH_4_ mixing ratio of 0.2–0.8 ppbv and three spikes that are an order of magnitude larger (Webster *et al.*, 2015, see Fig. 1a, b for details). These findings suggest that at least two types of CH_4_ emission are at work: a constant emission responsible for the background level and a mechanism for producing spikes. This discovery may come to be seen as one of the “Eureka moments” of the first half-century of robotic exploration of Mars, which began with the first success of the US spacecraft Mariner 4 in 1965. These intriguing findings compel a new era of Mars and astrobiological research to explain the existence and variability of CH_4_ in the martian atmosphere.

The TLS instrument in the Sample Analysis at Mars suite on the Curiosity rover has been making measurements for 3 years of the near-surface atmosphere of Mars at Gale crater. The TLS uses an infrared laser with a wavelength of 3.27 μm to scan over the R(3) spectral lines at ultrahigh spectral resolution. This is done in two measurement modes: a direct ingest with a typical uncertainty of 2 ppbv and an enriched method with a typical uncertainty of ∼0.1 ppbv, achieved by scrubbing out carbon dioxide during a slow fill of the sample cell.

In early measurements using only the direct ingest method, CH_4_ amounts were typically ∼1 ppbv (Webster *et al.*, 2013), except during a 2-month period when, for four sequential measurements, high mixing ratios of around 7 ppbv were observed, only to subsequently, and suddenly, disappear. This spike of high CH_4_ (Webster *et al.*, 2015), observed in northern hemisphere springtime, was attributed to a small local source. Daytime/nighttime differences in wind fields indicated a source to the north, most likely inside Gale crater. One Mars year later, the high values did not return, and a seasonal effect on the 10 × enhancement was therefore ruled out. A recent study of the CH_4_ observations from a variety of sources, including Curiosity, concluded that a cometary source of the CH_4_ (Kress and McKay, 2004; Fries *et al.*, 2016) was highly unlikely (Roos-Serote *et al.*, 2016).

Using the CH_4_ enrichment method, which provides sensitivity 23 times that of the direct ingest technique and typical uncertainties of 0.1–0.2 ppbv, the “background” low levels have been studied for nearly two consecutive martian years. Measurements thus far reveal an intriguing seasonal behavior (with the mixing ratio ranging from 0.2 to 0.8 ppbv; see Fig. 1b) that appears to correlate not with pressure, but with UV radiation. Models using expected amounts of infalling meteoric material and interplanetary dust, with a specified organic content and UV CH_4_ production efficiency, predict mean background levels of about 2.5 ppbv, some five times larger than that observed by TLS. Also, the magnitude of the range of TLS observations—from 0.2 to 0.8 ppbv—is much larger than that predicted for CH_4_ assuming its currently accepted long lifetime of ∼300 years, which far exceeds the global atmospheric mixing time of a few weeks to 1 month. Therefore, the TLS background measurements also imply that unknown loss (and source) processes are at work. Heterogeneous chemistry involving martian dust is a candidate that will be explored in laboratory studies.

## 3. Lifetime of CH_4_

Both the variability of the CH_4_ background and the spikes hint at a rather short lifetime of about one martian season, which conflicts with the atmospheric lifetime estimated using known gas-phase chemistry, summarized as follows.

The primary fate of CH_4_ on Mars is oxidation to CO_2_ and H_2_O:

\begin{align*}
{ \rm{C}}{{ \rm{H}}_4}  + { \rm{ OH}} \to { \rm{C}}{{ \rm{H}}_3}  + {{ \rm{H}}_2}{ \rm{O}}
\end{align*}

\begin{align*}
{ \rm{C}}{{ \rm{H}}_3}  + {{ \rm{O}}_2}  + { \rm{M}} \to { \rm{C}}{{ \rm{H}}_3}{{ \rm{O}}_2}  + { \rm{M}}
\end{align*}

\begin{align*}
{ \rm{C}}{{ \rm{H}}_3}{{ \rm{O}}_2}  + { \rm{H}}{{ \rm{O}}_2} \to { \rm{C}}{{ \rm{H}}_3}{ \rm{OOH}}  + {{ \rm{O}}_2}
\end{align*}

\begin{align*}
{ \rm{C}}{{ \rm{H}}_3}{ \rm{OOH}}  + h {\upnu} \to { \rm{C}}{{ \rm{H}}_3}{ \rm{O}}  + { \rm{ OH}}
\end{align*}

\begin{align*}
{ \rm{C}}{{ \rm{H}}_3}{ \rm{O}}  + { \rm{O}} \to {{ \rm{H}}_2}{ \rm{CO}}  + { \rm{OH}}
\end{align*}

\begin{align*}
{{ \rm{H}}_2}{ \rm{CO}}  + h {\upnu} \to {{ \rm{H}}_2}  + { \rm{CO}}
\end{align*}

\begin{align*}
{ \rm{CO}}  + { \rm{ OH}} \to { \rm{C}}{{ \rm{O}}_2}  + { \rm{H}}
\end{align*}

\begin{align*}
{ \rm{H}}  + {{ \rm{O}}_2}  + { \rm{M}} \to { \rm{H}}{{ \rm{O}}_2}  + { \rm{M}}
\end{align*}

\begin{align*}
{ \bf{Net :}}\;{ \rm{C}}{{ \rm{H}}_4}  + {{ \rm{O}}_2}  + { \rm{O}} \to { \rm{C}}{{ \rm{O}}_2}  + {{ \rm{H}}_2}{ \rm{O}}  + {{ \rm{H}}_2}.
\end{align*}

where M represents a third body (primarily CO_2_) in the ambient atmosphere.

The first reaction listed in the above oxidation scheme has a high activation energy, resulting in a long gas-phase lifetime of ∼300 years for CH_4_ (Summers *et al.*, 2002; Atreya *et al.*, 2007). For a discussion of this chemistry, which is well known in the terrestrial atmosphere, the reader is referred to Chapter 10 of Yung and DeMore (1999) and to Fig. 2a from Hu *et al.* (2012), based on the classic work of McElroy and Donahue (1972). To explain the rapid changes in CH_4_, as reported by Webster *et al.* (2015), the lifetime has to be shorter than 1 year (Lefèvre and Forget, 2009), which is incompatible with the standard working models of the chemistry of the martian atmosphere (Nair *et al.*, 1994; Chapter 7 of Yung and DeMore, 1999; Yung *et al.*, 2010). Nonstandard chemistry such as an enhanced source of H_2_O_2_, proposed by Atreya *et al.* (2007), is unlikely because the hypothetical oxidants will also oxidize CO and H_2_ in the martian atmosphere, and there is currently no evidence for additional destruction mechanisms for these species.

**FIG. 2.**
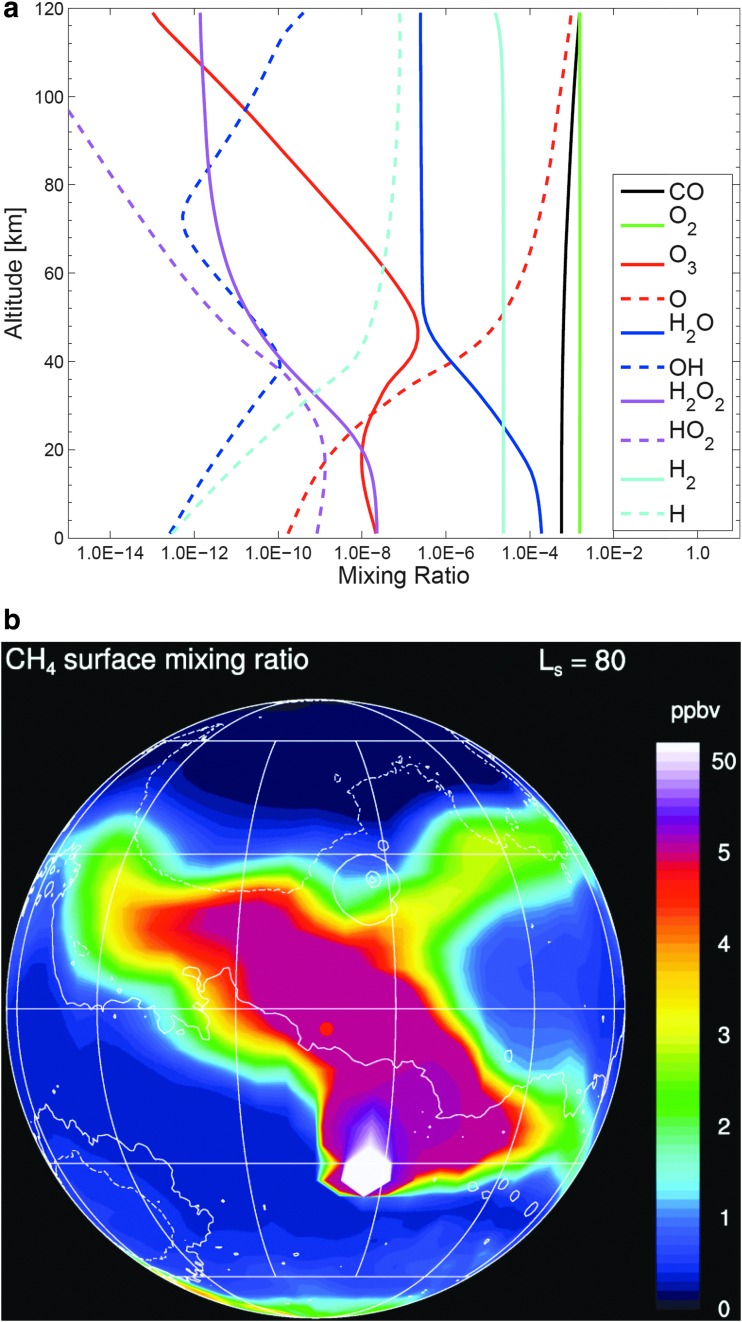
**(a)** Mixing ratios of minor species in the atmosphere of Mars (Hu *et al.*, 2012). **(b)** Simulation of CH_4_ with a source of 75,000 t/year at the Martz crater at *L*_s_ = 80° and a lifetime of 1 month. Based on a model by Lefèvre and Forget (2009).

A potential explanation for the observed seasonal variability of CH_4_ on Mars, without altering the basic atmospheric chemistry, is physical and chemical sequestration in the soil (Gough *et al.*, 2011; Jensen *et al.*, 2014; Hu *et al.*, 2016). In this case, the CH_4_ from the atmosphere is temporarily stored in the soil and later released to the atmosphere. This scenario produces a short lifetime for CH_4_ without actually destroying it. Presumably, seasonality should be inherent in such a process. Hence, such mechanisms might explain the background methane, but they cannot account for the methane spikes. According to Jensen *et al.* (2014), CH_4_ is removed when silicate grains form covalent bonds with CH_4_ via the following reaction:

\begin{align*}
{ \rm{Si}}  + { \rm{C}}{{ \rm{H}}_4} \to { \rm{Si}}  -  { \rm{C}}{{ \rm{H}}_3}  + { \rm{H}}.
\end{align*}

Nuclear magnetic resonance analysis indicated the formation of Si–CH_3_ bonds, which remain intact to at least 250°C (Jensen *et al.*, 2014).

The reverse reaction, releasing CH_4_ back to the atmosphere, has not been studied in the laboratory. However, we envision reactions such as the following:

\begin{align*}
{ \rm{Si}}  -  { \rm{C}}{{ \rm{H}}_3}  + { \rm{H}} \to { \rm{Si}}  + { \rm{C}}{{ \rm{H}}_4}
\end{align*}

\begin{align*}
{ \rm{Si}}  -  { \rm{C}}{{ \rm{H}}_3}  + { \rm{H}}{{ \rm{O}}_2} \to { \rm{Si}}  + { \rm{ C}}{{ \rm{H}}_4}  + { \rm{ }}{{ \rm{O}}_2} ,
\end{align*}

where the H and HO_2_ radicals are readily available on Mars from the photolysis of H_2_O and subsequent reactions:

\begin{align*}
{{ \rm{H}}_2}{ \rm{O}}  + h {\upnu} \to { \rm{H}}  + { \rm{OH}}
\end{align*}

\begin{align*}
{ \rm{H}}  + { \rm{ }}{{ \rm{O}}_2}  + { \rm{M}} \to { \rm{H}}{{ \rm{O}}_2}  + { \rm{M}}
\end{align*}

\begin{align*}
{ \rm{O}}  + { \rm{ OH}} \to {{ \rm{O}}_2}  + { \rm{H}} ,
\end{align*}

From a standard photochemical model of Mars, we can estimate the concentrations of H and HO_2_ at the surface of Mars to be as follows:

\begin{align*}
\left[ { \rm{H}} \right] { \rm{ }} \sim {10^4} { \rm{c}}{{ \rm{m}}^{ - 3}}
\end{align*}

\begin{align*}
\left[ {{ \rm{H}}{{ \rm{O}}_2}} \right] { \rm{ }} \sim {10^7} { \rm{c}}{{ \rm{m}}^{ - 3}}.
\end{align*}

We should point out that heterogeneous reactions (reactions between gas and solid reactants) have been proposed for Mars on the basis of laboratory studies (Anbar *et al.*, 1993).

Dynamical transport provides another indicator of the lifetime of CH_4_. Lefèvre performed dynamical simulations based on the model of Lefèvre and Forget (2009) to investigate 10 individual CH_4_ source locations (Apollinaris Patera, Gale crater, Elysium Mons, Lybia Montes, Martz crater, Medusae Fossae, Nier crater, Nili Fossae, Orcus Patera, and Tyrrhena Patera) over a range of source strengths and atmospheric lifetimes. As expected, scenarios involving 300-year lifetimes were far from compatible with the observed temporal pattern. Approximating the ∼7 ppbv TLS-observed spike required a lifetime of 1 month with a source strength of 75,000 t/year of CH_4_ (5 × 10^9^ mol/year) (Fig. 2b). For example, the regolith in Gale crater could adsorb methane when dry and release this methane to the atmosphere if deliquescence or a thin film of water/rock reactions occurs. A large but not prohibitive adsorption coefficient is required in this mechanism.

## 4. Geology and Geochemistry Relevant to Life on Mars

How life might emerge on a terrestrial planet such as Mars is far from settled. There are a multitude of ideas, each with their own intriguing pros and unresolved cons. They have quite a broad range: from the “primordial soup” hypothesis, encouraged by prebiotic chemistry experiments simulating a wet reducing atmosphere shocked by lightning (Miller, 1953), to the “clay life” hypothesis, which posits that life began as self-replicating minerals (Cairns-Smith and Hartman, 1986; Hartman, 1998), to the submarine alkaline hydrothermal vent theory (Russell and Hall, 1997), and to the “global chemical reactor” hypothesis, an intriguingly holistic approach that calls on contributions from nearly every aspect of a terrestrial planet (Stüeken *et al.*, 2013). In a comprehensive treatise, “The Origin and Nature of Life on Earth: The Emergence of the Fourth Geosphere,” Smith and Morowitz (2016) argue that the emergence of life followed a path of least resistance along the “long arc of planetary disequilibrium,” involving the atmosphere, oceans, and a dynamic mantle.

**FIG. 3.**
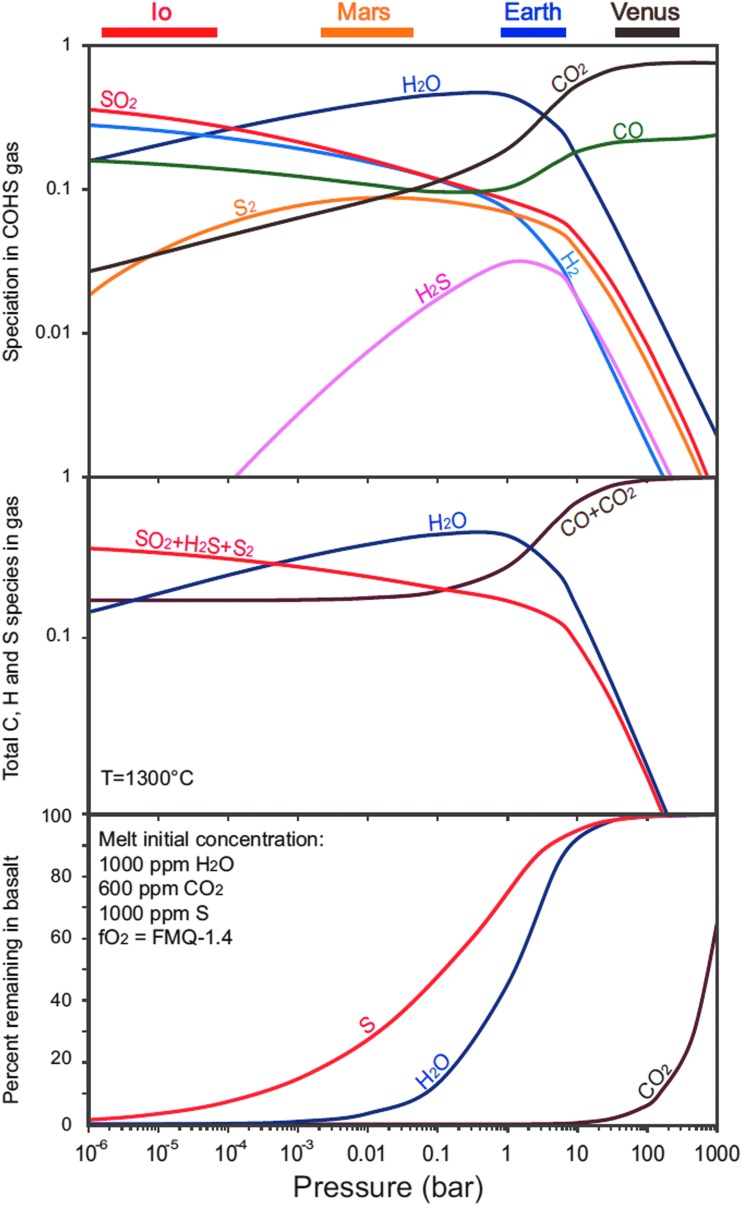
Global chemical trends of basalt degassing at pressures ranging from 1000 to 10^−6^ bar, encompassing subaerial venting conditions expected to prevail on telluric bodies. The total volatile abundances in the basalt are 600 ppm CO_2_, 1000 ppm H_2_O, 1000 ppm S. Taken from Gaillard and Scaillet (2014).

Metabolic processes that generate CH_4_ share certain similarities with—and, indeed, may have heritage in—certain abiotic geochemical pathways. Thus, for the purpose of this study, we consider life through the lens of its metabolic properties in the context of a planetary redox system. Our current knowledge indicates the following general characteristics of life: (1) it utilizes either photons or reduced compounds to drive redox reactions (involving oxidants such as perchlorate, sulfate, nitrate, and ferric iron) that fuel life's processes (Marlow *et al.*, 2014; Luo *et al.*, 2015); (2) it involves disequilibrium (free energy) conversions, much of them across cellular membranes and especially involving redox gradients (Shock, 1992; Russell and Hall, 1997); (3) it exploits Brownian motion, limiting its operations to −20°C and 120°C (Rivkina *et al.*, 2000; Takai *et al.*, 2008; Branscomb *et al.*, 2017); (4) it involves multiple feedbacks; and (5) it generates a myriad of mineral types beyond those of the initial geochemical inventory (Hazen *et al.*, 2008). Given these terms of reference, it behooves us to attempt to understand the still disputed initial redox states of Mars. H_2_, and possibly CH_4_, would have comprised a portion of the volatile flux from what was once a planet with low oxygen and high H_2_ fugacities (McSween *et al.*, 2009; McSween and Huss, 2010). However, did Mars always have a CO_2_ atmosphere? According to Gaillard and Scaillet (2014), magmatic degassing on Mars might have favored CO rather than CO_2_ as the dominant gas (Fig. 3). How did the atmosphere evolve, then, to its present condition, and over what pressures did it do so (Hu *et al.*, 2015; Ehlmann *et al.*, 2016)? Gaillard and Scaillet (2014) suggest that the composition of gases outgassed on Earth might have been significantly different from that on Mars (Fig. 3).

**FIG. 4.**
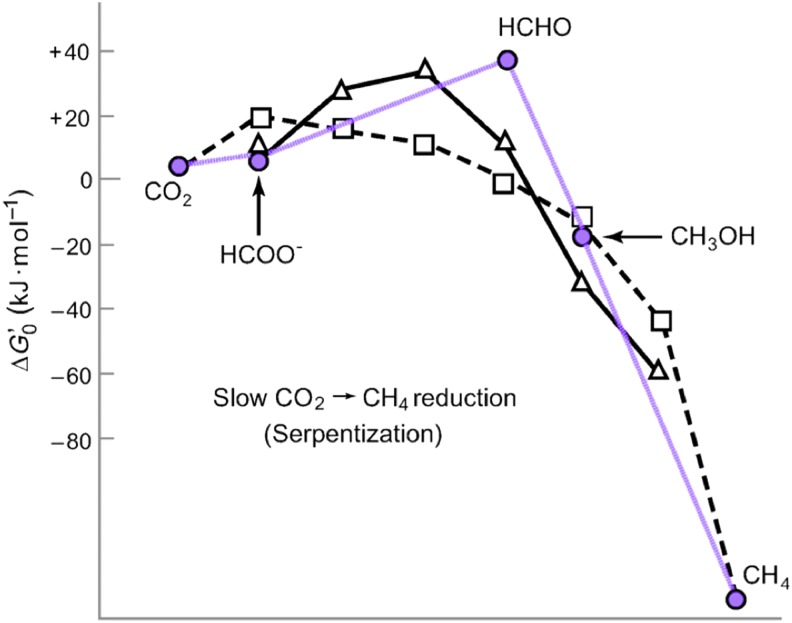
Free-energy profile of a hydrothermal pathway (in purple) to methane (Seewald *et al.*, 2006) is contrasted with the reduction profiles of the acetogenic bacteria (triangles) and methanogenic archaea (squares); both biological mechanisms use the acetyl coenzyme-A pathway. We can think of the geochemical pathway as a chemical siphon while the much more rapid biochemical pathways are driven by chemiosmosis over the intermediates formate and formaldehyde (or the formyl group). Adapted from Maden (2000).

Two processes involving the transformation of electrochemical energies through charge separation (across a membrane) constitute the fundamental sources of free energy fueling all known life; these are oxidative phosphorylation and electron bifurcation (Mitchell, 1975; Xia *et al.*, 2007; Schoepp-Cothenet *et al.*, 2012; Lubner *et al.*, 2017). In this respect, the planet acts as a battery providing electrical power, and a biological cell is analogous to a hydrogen burning fuel cell (Mitchell, 1967). The insides of prokaryote cells are also crowded and well structured, consisting of relatively electron-rich organic molecules that render them slightly alkaline, whereas their exteriors are generally more oxidized and acidic (Ellis, 2001; Lane, 2017). Thus, aqueous geochemistry in the former and biochemistry in the latter are both of a proton motive nature (Harold, 2001; Martin and Russell, 2007; Russell, 2007). The particular prokaryotic process that reduces carbon dioxide to CH_4_ is achieved by the methanogenic archaea. The abiotic pathway discussed in the next paragraph has to overcome thermodynamic barriers (comprising the two intermediates: formate, HCOO^−^, and formaldehyde, HCHO) and therefore prohibitively slow in the absence of catalysts (Fig. 4). For both the biotic and abiotic processes, the reaction rolls downhill in energy after formaldehyde formation, progressing toward a methyl group and thence to the stable CH_4_ molecule, away from the back reaction to formate and CO_2_ (Fig. 4). In other words, the hydrogenation reactions are “pulled” toward CH_4_ in a process that can be likened geochemically to siphoning (Russell and Hall, 2009). The biological pathway facilitates the process by way of the acetyl coenzyme A pathway, the most ancient of biochemical pathways (Fuchs, 1989). In this case, the same kinetic impediments to methanogenesis are cleared, although much more rapidly, with the energy provided by the proton motive force (Fig. 4).

Geochemical (abiotic) processes, generally known as FTT reactions, utilize H_2_ produced by serpentinization or other sources, such as the radiolysis of water (Sherwood Lollar *et al.*, 2014), and magma degassing under reducing conditions to reduce CO_2_. The breakdown of iron-bearing primary minerals, such as olivine in the serpentinization process, allows electrons to be transferred from ferrous iron to H_2_ as ferrous iron is oxidized to form magnetite (Abrajano *et al.*, 1990; McCollom and Bach, 2009; Holm *et al.*, 2015; McCollom and Donaldson, 2016).

\begin{align*}
& 6 { \rm{M}}{{ \rm{g}}_{1.5}}{ \rm{F}}{{ \rm{e}}_{0.5}}{ \rm{Si}}{{ \rm{O}}_4}  \, +  \,7{{ \rm{H}}_2}{ \rm{O}} \to 3 { \rm{M}}{{ \rm{g}}_3}{ \rm{S}}{{ \rm{i}}_2}{{ \rm{O}}_5}{ \left( {{ \rm{OH}}} \right) _4}   +  \,{ \rm{F}}{{ \rm{e}}_3}{{ \rm{O}}_4}  \, +  \,{{ \rm{H}}_2} \\ & \quad\quad { \rm{olivine}} \quad \quad \quad \quad \quad\quad\quad \quad \quad{ \rm{serpentine}} \quad \;\;{ \rm{magnetite}} \\
\end{align*}

In a second, separate step, an FTT reaction can take place through which H_2_ reduces carbon dioxide to CH_4_. Thus, given a source of CO_2_ and H_2_, as we have seen, it is possible to form CH_4_. CH_4_ production is possible and relatively rapid, even at low temperatures, via gas-phase FTT reactions (both CO_2_ and H_2_ in the gaseous phase, *e.g.*, in dry or unsaturated rocks), in the presence of metal catalysts (heterogeneous catalysis; Etiope and Ionescu, 2015).

\begin{align*}
{ \rm{C}}{{ \rm{O}}_2}  + 4{{ \rm{H}}_2} \to { \rm{C}}{{ \rm{H}}_4}  + 2{{ \rm{H}}_2}{ \rm{O}}
\end{align*}

FTT reaction is feasible only in dry, *gas-phase* conditions, because dissolved phases of CO_2_ and H_2_ are not chemisorbed on the metal catalyst and the reaction does not proceed toward CH_4_ and H_2_O. This implies that CH_4_, an electron donor for prebiotic chemistry, can be obtained in the absence of water. This can occur in rocks particularly enriched with metals that can act as Sabatier catalysts, such as chromium- and ruthenium-enriched chromitites, for example, the Chassigny meteorites, which likely occur in several regions of Mars (Oehler and Etiope, 2017). After CH_4_ is generated, it may migrate toward rocks containing water, where biological processes can operate.

Aside from redox conditions enabling CO_2_ hydrogenation, we must also ask if early Mars was habitable with respect to the presence of liquid water. An Earth-like environment with open oceans is probably unlikely. Instead, we envision a colder, more arid atmosphere/hydrosphere with episodes of surface water (Ehlmann *et al.*, 2011). Driven by obliquity cycling and early loss of water to space, arid conditions and episodically icy highlands may have been the norm (Lammer *et al.*, 2013; Wordsworth, 2016). Emergence of life under icy conditions could be possible (Russell *et al.*, 2014). More clement episodes might occur during periods of enhanced volcanism or glaciation/deglaciation cycles resulting from climate feedbacks (Batalha *et al.*, 2016; Kite *et al.*, 2017). Lakes certainly existed for intervals of thousands of years (Grotzinger *et al.*, 2014, 2015), and mineralogical evidence points to persistent, long-lived groundwater reservoirs, while surface waters were more episodic (Ehlmann *et al.*, 2011). In the Gale crater region, the ancient aqueous environment was characterized by neutral pH, low salinity, and variable redox states of both iron and sulfur species, and key biogenic elements—carbon, hydrogen, oxygen, sulfur, nitrogen, and phosphorus—were measured. Deuterium/hydrogen isotope ratios from ancient clays in a Gale lake deposit (Mahaffy *et al.*, 2015) indicate that a global equivalent layer of water of 100 to 150 m in depth was present at the time of sediment accumulation (Grotzinger *et al.*, 2015). Hydrogen gas production was enabled by diagenetic, groundwater reactions to form magnetite and saponite from olivine (Bristow *et al.*, 2015). Under these circumstances, life could have emerged.

If life did emerge on Mars, there are at least two reasons for it to have gone underground and use chemical reactions from H_2_ and CH_4_ for energy. First, the loss of a thicker early Mars atmosphere may have led to the loss of its associated greenhouse effect, resulting in a colder, more arid and more radiation-exposed surface (Hu *et al.*, 2015). Second, the loss of H_2_ by escape from the top of the atmosphere may have left behind an increasingly oxidized surface environment. Early methanogens on Mars, which might have emerged in the early, more reducing environment, would have migrated to the subsurface in ways similar to the migration of terrestrial anaerobes to the “deep biosphere.” Methanogens and methanotrophs could exist today if the subsurface of Mars had water, which some speculate (Grimm *et al.*, 2017).

## 5. Detection of CH_4_ Seepage

It is widely accepted that the occurrence of CH_4_ in the martian atmosphere may imply the presence of active geological sources, that is, gas emission structures in the martian soil and subsoil. In other words, gas seepage, a process well known on Earth, should exist on Mars. An extensive literature exists on terrestrial seepage; definitions, geological and geochemical processes, implications, and a literature review are summarized in Etiope (2015). We also refer the reader to Oehler and Etiope (2017) for a recent review of processes that can possibly generate methane seepage on Mars and identification/detection methods. The following is a brief summary of some relevant points.

Seepage on Mars can be revealed in specific surface manifestations in association with faults and fractured rocks, analogous to those observed on Earth. Gas seepage structures may include small circular depressions along faults, polygonal fractures, mounds, and mud volcanoes. Similar geological structures have already been observed in many areas on Mars (Oehler and Allen, 2010; Etiope *et al.*, 2011b, 2013), but their actual gas-bearing role is unknown (Wray and Ehlmann, 2011). Gas seepage on Mars, as on Earth, can also be in the form of invisible diffuse exhalation from the ground (microseepage; see Etiope, 2015). Such visible or invisible seepages may develop in different geological settings on Mars, either sedimentary basins or basaltic, mafic, and ultramafic terrains, depending on the potential origin of CH_4_. After CH_4_ is formed, it may accumulate in porous and permeable rocks or clathrates acting as reservoirs. From these storage reservoirs, the gas may seep to the surface, preferentially along permeable pathways, such as faults and fractured rocks. For details on the main concepts of hypothetical CH_4_ production, storage, and seepage on Mars, see Oehler *et al.* (2005), Oehler and Allen (2010), Etiope *et al.* (2011a), Etiope *et al.* (2013), Etiope (2015), Oehler and Etiope (2017), and Etiope (2018).

How can we detect seepage on Mars? Based on analogy to the Tekirova ophiolites in Turkey where substantial amounts of CH_4_ are produced by active low-temperature (<140°C) serpentinization and abiotic methanation, flux rates of several mg/(m^2^·day) could occur in martian rocks (Etiope *et al.*, 2013). However, measurements of CH_4_ in the atmosphere taken 1 m above the ground, such as those performed by the Curiosity rover, may not be effective in revealing any seepage. As on Earth, the processes transporting CH_4_ to the surface and consuming CH_4_ en route may prevent its detection in the atmosphere even several centimeters above ground. These en route alterations can be important for Mars, where oxidative regolith CH_4_ chemistry could effectively remove methane. Opportune procedures and techniques must be adopted to detect seepage on Mars. One possibility could be to consider available surface gas geochemical techniques used on Earth, developed by petroleum geologists and geochemists, which allow the discovery of seepage and related underground hydrocarbon reservoirs all over the world using soil gas sampling, accumulation chambers, downhole analysis, and observations of surface mineralogical alterations (see Etiope, 2015; Oehler and Etiope, 2017, and references therein). In summary, geologic terrains characterized by regional faulting or including apparent mud volcanoes are the best places, according to present knowledge, to search for CH_4_ seepage on Mars, preferably above or near regions with olivine-bearing or sedimentary rocks. Extensive Earth-based experience in seepage sampling, detection, and analysis provides valuable guidance for future Mars exploration and technology development.

## 6. ExoMars TGO

The ExoMars program, a European Space Agency–Roscosmos cooperation with some NASA contributions, consists of two missions, one that was launched in 2016 and a second one that is scheduled for launch in 2020. The 2016 mission includes an orbiting satellite, the TGO, dedicated to the study of atmospheric trace gases to acquire information on possible ongoing geological or biological processes. This instrument is currently beginning normal science operations. Schiaparelli, an entry, descent, and landing demonstrator module, was intended to demonstrate the European Space Agency's technical prowess in safely landing modules on the surface of Mars, but failed at landing in November 2016. The 2020 mission is scheduled to deliver a 300 kg class rover and an instrumented landing platform to the martian surface using a landing system developed by the Russian Space Agency, Roscosmos.

The scientific objectives of the payload aboard the TGO and on the 2020 surface platform and rover are as follows: (1) to search for signs of past or present life on Mars; (2) to investigate how the water and geochemical environments vary, that is, by characterizing the distributions of relevant molecules in the atmosphere, on and near the surface, or as a function of depth in the shallow subsurface; (3) to investigate martian atmospheric trace gases and their sources; (4) to study the surface environment and identify hazards to future crewed missions to Mars; and (5) to investigate the planet's subsurface and deep interior to better understand the evolution and habitability of Mars. If successful, TGO will provide a major advance in the characterization of Mars' atmospheric chemistry.

In particular, the presence of two spectroscopic suites, Nadir and Occultation for Mars Discovery (NOMAD; Vandaele *et al.*, 2015) and Atmospheric Chemistry Suite (ACS; Korablev *et al.*, 2015), will help TGO cover several distinct areas:

(1) Analyzing the present-day chemical composition of the martian atmosphere through the detection of a broad suite of trace gases and key isotopes. Covering 14 UV, visible, and IR spectral ranges, NOMAD and ACS will ensure that a large number of species are detectable, such as CO_2_ (including ^13^CO_2_, ^17^OCO, ^18^OCO, and C^18^O_2_), CO (including ^13^CO and C^18^O), H_2_O (including HDO), NO_2_, N_2_O, O_3_, CH_4_ (including ^13^CH_4_ and CH_3_D), C_2_H_2_, C_2_H_4_, C_2_H_6_, H_2_CO, HCN, OCS, SO_2_, HCl, HO_2_, and H_2_S. The high resolution of the NOMAD IR channels will provide highly resolved spectra, allowing unambiguous separation of absorption lines and thus ensuring a high sensitivity for the search of trace gases (see Robert *et al.*, 2016, for a detailed analysis of the sensitivity of the NOMAD instrument).
(2) Extending trace gas detection to the upper atmosphere to constrain atmospheric escape processes relating the present-day atmosphere to its past and future evolution. Simultaneous vertical profile measurements of H_2_O, HDO, and atmospheric temperature will help investigate escape processes and evaluate upward fluxes and vertical diffusion up to the exobase.
(3) Understanding the chemistry to constrain the origin of CH_4_ (*i.e.*, geophysical, exogenous, or biological) and destruction processes. NOMAD will contribute to solving the current question of the existence and persistence of martian CH_4_ by providing unequivocal measurements of its presence and variability, and it will clarify the processes related to its origin and destruction through the simultaneous detection of CH_4_ isotopologues and higher hydrocarbons such as C_2_H_4_ or C_2_H_6_.
(4) Studying gases related to possible ongoing geophysical and volcanic activity on Mars. Major gas releases associated with volcanic outgassing are expected to be sulfuric, that is, SO_2_ and H_2_S, which lie in the sensitivity range of NOMAD. This might help to distinguish whether CH_4_ is associated with either low-temperature (serpentinization, radiolysis of water, and subsequent Fischer-Tropsch-type reactions) or high-temperature magmatic processes.

The 2020 ExoMars rover will carry a comprehensive suite of instruments dedicated to geology and exobiology research. It will travel several kilometers searching for places potentially harboring signs of past or present life on Mars. Materials will be collected from the subsurface down to a depth of 2 m (in ideal situations) through the use of a dedicated drill and the powders examined *in situ* with the science payload. The descent platform will also carry payload instruments to study the martian surface environment.

The ExoMars rover will search for two types of life-related signatures, morphological and chemical, complemented by a detailed description of the geological context. Morphological biosignatures can be preserved on the surface of rocks, such as microbially mediated deposition of sediments, fossilized microbial mats, or stromatolitic mounds. Their study requires imaging systems capable of submillimeter resolution. Chemical biosignatures include chemical gradients, minerals, and isotopes preserved in the geologic record.

## 7. Habitability and Biosignatures

In the past few decades, the definition of habitability has changed dramatically as a result of the discovery of abundant life in the depths of Earth's oceans (around hydrothermal vents), the remarkable ability of extremophiles to tolerate conditions not imagined 50 years ago, and the discovery of abundant life in the terrestrial subsurface of our own planet. The old notion of “liquid water at the surface” has disappeared as the factor of importance, to be replaced by “a planet or moon that can supply the elements needed for life to emerge and thrive, be it on the surface, in the subsurface, or in a sub-ice-covered ocean.” Thus, Mars is no longer necessarily beyond the habitable zone, and certainly the moons of the jovian and saturnian systems (*e.g.*, Europa, Enceladus, Titan), located far from the “habitable” (a.k.a. Goldilocks) zone as defined in the past, are now reasonable candidates for habitable sites (Russell *et al.*, 2014). We have moved from a habitable zone to a set of sites that meet the “habitable criteria”—a much different concept.

So, what are the criteria of habitability?

(1) *Presence of a solvent in which electrochemical transformations can occur*. An essential feature of life is the invention and use of enzymes to harvest and transform free energy from a geochemical environment where processes are otherwise slow or unworkable. Often thought of merely as “life's solvent,” water is indispensable in that it acts as a shell to all proteins, and is necessary for their interaction with their substrates (Ball, 2008). Moreover, all known biochemical transformations (disequilibria conversions) are “biased” diffusional processes only known to operate in the extreme low Reynolds number regime offered by aqueous media (Astumian, 2006). This solvent would not have to be at the surface, but if life were global, the solvent would have to be widespread. Recent discovery of widespread excess ice in the subsurface (Boynton *et al.*, 2002; Mitrofanov *et al.*, 2002; Tokar *et al.*, 2002; Bramson *et al.*, 2015; Stuurman *et al.*, 2016; Dundas *et al.*, 2018) suggests that Mars is water rich.
(2) *Presence of sources of free energy to support life*. On Earth, we have abundant solar energy and abundant geochemical energy in the form of redox couples, both of which support terrestrial life. Chemical energy derived from photolysis of CO_2_ and H_2_O is available on Mars (Weiss *et al.*, 2000). There is a potential for photo- and biochemical generation of organic molecules from H_2_O and CO_2_ on Mars, although no evidence has been forthcoming to date (Weiss *et al.*, 2000). There are other energy sources (magnetic, heat, nuclear, and wind), but as far as we know, no earthly life (other than human) has solved the problem(s) of metabolizing any of these. Recent reports of microbes that live on electricity are an interesting addition to the redox style of life (Rowe *et al.*, 2015), and could offer connections to life forms that utilize magnetism. The point is habitability will require available sources of free energy (*i.e.*, appropriate disequilibria), and these should be assessed at any site where life is suspected to be present. Recent studies on the role of radiolysis not only in producing H_2_ but, via indirect oxidation of sulfide minerals, in producing sulfate as well, demonstrate novel mechanisms by which both essential electron donors (CH_4_, H_2_) and electron acceptors (sulfate) can be provided to sustain subsurface chemolithotrophic communities, even in otherwise oligotrophic crystalline rocks billions of years in age (Lin *et al.*, 2006; Li *et al.*, 2016).
(3) *Presence and availability of elements needed for life.* Life as we know it is unique in its elemental composition—no known abundant mineral is composed of the same group of elements. With a few exceptions (such as Si-rich diatoms), life on Earth is composed of the same elements, in roughly the same ratios. While there is no need to argue that life elsewhere would be composed of the same elements, it is reasonable to suppose that life at another site will have its own composition that allows it to harvest energy, grow, reproduce, and evolve. Nevertheless, given that life exploits chemical and chemiosmotic energy, it would be hard to imagine it operating in the absence of Fe and Ni for hydrogenations and dehydrogenations, Mo/W for electron transfer, Co for methyl transfer, P for linking nucleotides and disequilibria transformations, and S for iron/sulfur clusters, and the cysteine/cystine redox cycle (Westheimer, 1987; Beinert, 2000; Nitschke and Russell, 2009; Nitschke *et al.*, 2013).

Mars offers a unique combination of conditions for acquiring understanding not attainable by experiments elsewhere. To put this another way, there are no true Mars analog environments on Earth. Mars has only a few millibar of atmosphere, primarily composed of CO_2_ with very little oxygen or nitrogen. It has little or no surficial liquid water and very little organic carbon as well. Lacking a thick atmosphere, it thereby bathes in damaging ultraviolet and ionizing radiation. Thus, while Mars is roughly similar to Earth in size (half the Earth's diameter and hence about 10% its mass), chemical composition, and distance from the Sun—making it a candidate for comparison and interpretation—it is sufficiently different to provide tests not attainable on Earth. Irrespective of whether life is or was ever present on Mars, there are a number of key astrobiological issues that have been and will continue to be addressed through the study of our sister planet. Geological evidence and models suggest that early Mars was more Earth like, with a magnetic field, an ocean, warmer ambient temperatures than at present, a denser atmosphere, and perhaps even some rudimentary plate tectonics. Just how long the martian environment remained with such clement conditions, and whether life might have evolved during that time, is one of the key astrobiological questions that might be answered by detailed studies of Mars. In the following paragraphs, we focus on a few key components of habitability concerning methane on Mars.

### 7.1. Source identification

The astrobiological relevance of CH_4_ on Mars pertains to the possible existence of life and/or habitability. If it can be established that CH_4_ is being injected into the martian atmosphere, and the rates can be deemed significant, then the planet is at least geochemically active and capable of H_2_—and eventually CH_4_—production. Given that both H_2_ and CH_4_ are common metabolites of life on Earth, the notion of extant life on Mars becomes a possibility. However, at the same time, production of H_2_ and CH_4_ can occur in water/rock reactions such as serpentinization and FTT reactions on Earth, although their kinetics as a function of temperature is still debated. While little is known about subsurface water flow, diffusion, and water/rock reactions on Mars (see recurring slope lineae observations possibly indicating the subsurface flow of water, Ojha *et al.*, 2015), if such processes occur, then H_2_ and/or CH_4_ could possibly be generated abiotically (Lyons *et al.*, 2005; Oze and Sharma, 2005; Onstott *et al.*, 2006). Certainly, fracture waters down to at least 3 km depth in the Precambrian Shields of Earth (including Archean rocks of circa 3–2.7 Ga) contain large quantities of reduced gases (mM concentrations of H_2_, CH_4_, and higher hydrocarbons; see Sherwood Lollar *et al.*, 2002, 2014).

A key to understanding the relationship of CH_4_ to the habitability of Mars (now or in the past) lies in the stable isotopic signature of any CH_4_ that is found (Allen *et al.*, 2006). On Earth, the carbon isotope ratio (*e.g.*, δ^13^C) in biologically formed CH_4_ is highly enriched in ^12^C relative to ^13^C by methane-producing archaea, yielding δ^13^C values of −60‰ or lighter (*i.e.*, more negative). While strongly ^13^C-depleted methane would be an exciting finding because it would suggest a causal indication of present or past microbial life on Mars, interpretations must be tempered, since equally strong fractionations are associated with abiotic methanogenic processes. Hence, δ^13^C signatures and fractionation factors alone are insufficient to differentiate sources (Sherwood Lollar *et al.*, 2006; McCollom *et al.*, 2010; Etiope, 2018). Any isotopic analysis of CH_4_ must be coupled with multiple lines of evidence, including stable isotopic signatures of the potential carbon sources (*e.g.*, CO_2_, CO) or additional products of hydrocarbon synthesis (*e.g.*, ethane, propane) and associated sulfur-bearing compounds or noble gases (Allen *et al.*, 2006; Etiope and Sherwood Lollar, 2013). By contrast, unfractionated CH_4_ will be less definitive; depending on the reservoirs, formation pathways, enzymes involved, rates of production and consumption, and other variables, there should be plenty of ways to produce unfractionated CH_4_ biologically as well as abiotically. Correction of δ^13^CH_4_ values caused by atmospheric reactive losses must also be taken into account (Nair *et al.*, 2005). In any case, it is clear that any well-documented large CH_4_ emissions would be a cause for excitement and further study.

### 7.2. Climatic habitability

In the past, at larger outgassing rates, reduced gases such as H_2_ and CH_4_ from the subsurface may have played a key role in the habitability on Mars. Atmospheres rich in H_2_ could supply the needed temperatures to sustain liquid water at the surface. Ramirez *et al.* (2014) demonstrate that an atmosphere containing 1.3–4 bar of CO_2_ and water, in addition to 5–20% H_2_, could have raised the mean surface temperature of early Mars above the freezing point of water. The source could have been volcanic outgassing from a more reduced early martian mantle. Even low levels of methane supply to the surface could provide needed warming, if CH_4_ is emitted in a punctuated manner (to replenish atmospheric CH_4_ with a finite subsurface inventory). For example, Wordsworth *et al.* (2017) show that methane and hydrogen produced following aqueous alteration of Mars' crust could have combined with volcanically outgassed CO_2_ to form transient atmospheres and that methane could have acted as a powerful greenhouse gas on early Mars due to CO_2_–CH_4_ collision-induced absorption in the critical 250–500 cm^−1^ spectral window region. Kite *et al.* (2017) suggest that methane clathrates released by obliquity cycles may episodically build up to atmospheric levels sufficient for intermittent, lake-forming climates, assuming methane clathrate initially occupied more than 4% of the total volume in which it is thermodynamically stable.

The preceding discussion of outgassing evokes the question of whether or not plate tectonics is a requirement for habitability. Ancient Mars was tectonically active, but any such activity has long since stopped. This puts the study of life's history on Mars in a unique place in terms of addressing this question. If life did arise on early Mars, was it impossible for it to continue in the absence of geological cycling of the elements, as occurs on Earth? Recent evidence from the tectonically quiescent 2.7 Ga cratons on Earth demonstrates that fracture propagation continues even in the absence of plate tectonics (Sleep and Zoback, 2007), and that hydrogen H_2_ (and CH_4_) production, as well as sulfate production, could continue on a geologic timescale even at low temperatures (Sherwood Lollar *et al.*, 2014; Li *et al.*, 2016). Such processes and timescales may provide relevant analogs for Mars, in addition to studies focused on high-temperature water–rock interactions on the relatively young (<200 Ma) marine seafloor.

## 8. Atmospheric Transport and Measurement Strategies

Understanding the distribution and fate of CH_4_ in the martian atmosphere—as well as identifying potential surface sources—relies heavily on our understanding of atmospheric transport on Mars. Atmospheric observations from Mars orbiters over the last two decades have contributed to a fairly comprehensive picture of the structure of the martian atmosphere. The characterization of the middle atmosphere (∼50–100 km altitude) has been greatly improved through recent observations by both the Mars Climate Sounder on the Mars Reconnaissance Orbiter, covering altitudes between the surface and ∼80 km (Kleinböhl *et al.*, 2009; McCleese *et al.*, 2010), and Spectroscopy for Investigation of Characteristics of the Atmosphere of Mars on Mars Express, covering altitudes between 70 and 130 km (Forget *et al.*, 2009). The upper atmosphere (>100 km) has been characterized during aerobraking with entry, descent, and landing density profile measurements (Withers and Smith, 2006). More recently, the Mars Atmosphere and Volatile Evolution (MAVEN) mission, with its focus on this atmospheric region, has been providing a wealth of new observations that will make significant contributions to our understanding of upper atmospheric dynamics and escape, indicating significant atmospheric mass loss in the last 4.5 Gyr (Jakosky *et al.*, 2017).

**FIG. 5.**
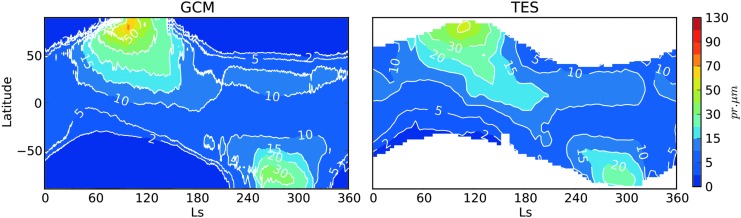
(Left) Column-integrated, zonal mean daytime water abundance for present-day Mars, modeled by the LMD MGCM. (Right) Column-integrated, zonal mean daytime water abundance from the MGS-TES instrument for MY 26. From Navarro *et al.* (2014b). LMD, Laboratoire de Météorologie Dynamique; MGCM, Mars general circulation model; MGS, Mars Global Surveyor; TES, Thermal Emission Spectrometer.

Numerical models are the primary means of representing our understanding of the martian atmosphere. Several state-of-the-art Mars general circulation models (MGCMs) for simulating atmospheric structure and dynamics are available in the community. These include the NASA Ames MGCM (Kahre *et al.*, 2015), the Laboratoire de Météorologie Dynamique (LMD) MGCM (Forget *et al.*, 1999; Navarro *et al.*, 2014b), and the Mars Weather Research and Forecasting (MarsWRF) General Circulation Model (Richardson *et al.*, 2007; Toigo *et al.*, 2012, among others). Figure 5 shows a comparison of the atmospheric water cycle by the LMD GCM to Mars Year 26 observations by the Thermal Emission Spectrometer aboard the Mars Global Surveyor (from Navarro *et al.*, 2014b). These results are consistent with more recent observational data (Tschimmel *et al.*, 2008; Smith *et al.*, 2009; Sindoni *et al.*, 2011) and other MGCMs as well. Additional MGCMs available in the community include the Geophysical Fluid Dynamics Laboratory (GFDL) MGCM (Wilson and Hamilton, 1996; Greybush *et al.*, 2012) and the Global Environmental Multiscale model (Daerden *et al.*, 2015).

**FIG. 6.**
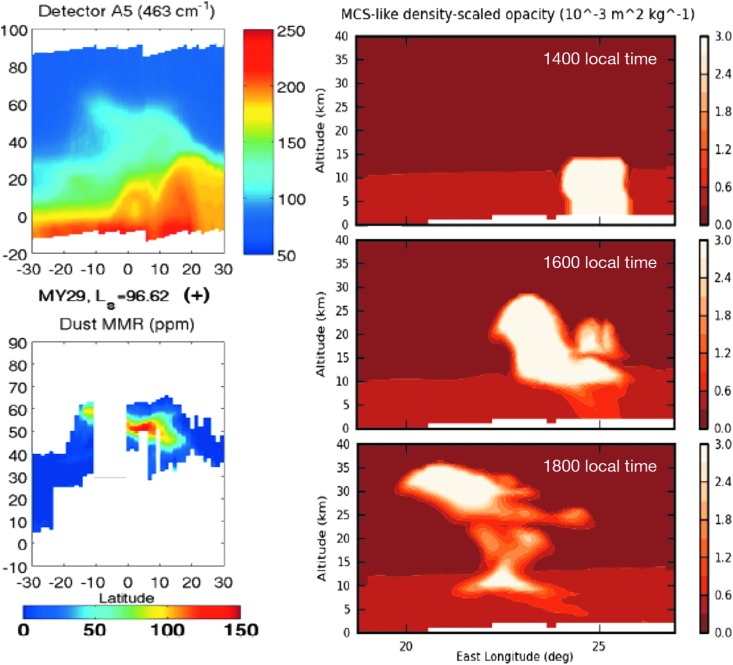
(Left): Limb radiances at 463 cm^−1^ measured by MCS (top) and dust mass mixing ratio retrieved from MCS measurements (bottom), exhibiting a detached dust layer at 50–60 km altitude over Tharsis (Heavens *et al.*, 2015). The density-scaled optical depth (d_z_τ) is related to the optical depth at 0.67 μm (dτ) by d_z_τ = −dτ/ρ, where ρ is atmospheric density in units of kg/m^3^. (Right): Mesoscale model simulation showing density-scaled dust opacity in a so-called rocket dust storm, in which dust can be lifted to altitudes 30–40 km within a few hours (Spiga *et al.*, 2013). MCS, Mars Climate Sounder.

While comparisons of available measurements of atmospheric structure and aerosol distribution with MGCMs show agreement in many aspects, they also reveal significant discrepancies (McCleese *et al.*, 2008; Forget *et al.*, 2009; McDunn *et al.*, 2010). Improvements in numerical models require the identification and quantification of the underlying dynamical and radiative processes that govern the martian atmosphere, as well as the chemical processes relevant to CH_4_ and other trace gases. Recent advances in our understanding of transport in the martian atmosphere include the recognition of the radiative influence of aerosols, and the significant impact large-scale dust storms have on atmospheric structure. However, water/ice clouds also exert a significant radiative influence on the atmosphere, mostly through absorption of infrared radiation emitted from the surface during the day. This radiative influence has a significant impact on atmospheric tides and increases the speed of the overturning meridional circulation (Madeleine *et al.*, 2012; Kleinböhl *et al.*, 2013; Steele *et al.*, 2014), leading to an increase in the transport of aerosol and trace constituents toward the poles. Also, the coupling between the dust and water cycles leads to radiative dynamic feedbacks that have only recently been recognized (Kahre *et al.*, 2015). Nevertheless, features such as detached dust layers in the middle atmosphere of Mars (Heavens *et al.*, 2011; Heavens *et al.*, 2015; see Fig. 6) and their variability (Heavens *et al.*, 2014) lack a comprehensive explanation and indicate that significant shortcomings in our understanding of atmospheric transport still exist. The absorption of sunlight by dust layers can lead to local atmospheric warming, which affects the buoyancy of air parcels. Daerden *et al.* (2015) identified such a solar escalator effect by tracking dust layers in the northern polar region. Due to the higher insolation, it is expected that such effects are more vigorous at middle and low latitudes. Simulations with mesoscale models suggest that local dust heating can lift dust layers by 10–20 km in altitude within a few hours (Spiga *et al.*, 2013; and Fig. 6). These processes are likely to play a major role in the vertical transport of aerosols and trace gases but are currently not well quantified or represented in MGCMs. Continued atmospheric observations targeted at understanding these processes and improvements in their representation in models are required to further our understanding of the dynamics of the martian atmosphere.

CH_4_ measurements on Mars have clearly demonstrated the critical need for enabling back-trajectory analyses. For instance, we cannot determine the source location of the TLS-observed CH_4_ plume because we do not have the ability to calculate back trajectories for Mars. Lefèvre and Forget (2009, with updates) presented preliminary studies in which their MGCM simulated ten putative source locations, all equally likely due to lack of observational constraints (see, *e.g.*, Fig. 2b). In addition, atmospheric circulation can significantly affect atmospheric chemistry.

A prerequisite for carrying out accurate back-trajectory analyses is a detailed knowledge and representation of the atmospheric state. On Earth, such knowledge is gathered by assimilating atmospheric data from radiosondes and satellites into general circulation models. The results of these assimilations (called “reanalysis”) are sufficiently accurate to be treated as the “true” atmospheric state. For accurate back-trajectory calculations, it is essential that this capability be developed for Mars, as free-running MGCMs do not represent the atmospheric state well enough to be applicable for studies at this level of detail. Initial efforts have been undertaken to assimilate temperature and/or aerosol distribution from spaceborne observations into MGCMs (Lee *et al.*, 2011; Greybush *et al.*, 2012; Navarro *et al.*, 2014a; Steele *et al.*, 2014). Assimilation models need to be run at sufficiently high spatial and temporal resolution to provide reanalyses of sufficient detail to be useful for back-trajectory calculations.

For this effort to be successful, suitable atmospheric observations must be available to be assimilated. This requires the continued operation of current assets, as well as the deployment of new assets. Assets deployed into Mars orbit need to measure atmospheric profiles of temperature or radiance, dust, water/ice, aerosol, and (ideally) water vapor. Measurements must have continuous global coverage on at least a daily basis to be useful for assimilation. Limb sounding in the thermal infrared can provide this kind of information with the required coverage from a low Mars orbit. Radio science measurements lack aerosol information and are unlikely to achieve the global coverage required to be suitable for assimilation. However, their highly accurate temperature measurements make them a good candidate for the validation of reanalyses. Another data set that might be suitable for assimilation is surface pressure obtained by a network of *in situ* sensors on the Mars surface, assuming that a sufficient number of nodes can be deployed with enough spatial coverage. This will require technological development.

If the short lifetime of CH_4_ on Mars suggested by previous measurements is confirmed by TGO, then a strong case can be made for an episodic release from sources. It will be important to capture episodic enhancements of CH_4_, as we have not yet discerned any predictable geographical or temporal pattern for such events. Therefore, CH_4_ measurements with global and quasicontinuous observational coverage are indispensable. TGO requires more than a month to attain quasiglobal coverage, which may not be sufficient.

One approach for achieving this goal is CH_4_ mapping from low Mars orbit with high spatial resolution. Measurement of reflected sunlight in the infrared in nadir/off-nadir geometry would be the preferred technique. The strongest CH_4_ lines are found at ∼3000 cm^−1^, which is also the band in which NOMAD targets its CH_4_ measurements (Robert *et al.*, 2016). Absorption measurements of these lines with high spectral resolution (∼0.1 cm^−1^) provide column measurements of CH_4_. An imaging spectrometer would be the instrument of choice to obtain both high spatial coverage and resolution. This could be obtained with instruments developed for remote sensing of column O_2_ and CO_2_ in Earth's atmosphere in a wavelength range from 0.7 to 2 μm (Crisp *et al.*, 2012). This technology is basically available, but would have to be adapted for CH_4_ measurements on Mars. The small absorption signal from the expected CH_4_ levels at Mars will likely require a cooled spectrometer and detector array to keep pixel sizes small and integration times short such that high spatial resolution can be achieved. We note that NOMAD's cooler for nadir viewing measurements was descoped (Vandaele *et al.*, 2015). The deployment of an instrument with a cooled spectrometer and detector array would hence provide a significant increase in sensitivity. The field-of-view of such an imaging spectrometer could be gimbaled in the cross-track direction to increase the swath width of the measurement and to allow targeted observations.

Interplanetary CubeSats and SmallSats may provide novel, low-cost means to deploy instrumentation for atmospheric and/or chemical studies from Mars orbit or on the martian surface. One approach is a CubeSat constellation in Mars orbit that could perform observations of atmospheric temperature, dust, and water/ice profiles. This could be achieved through thermal infrared limb sounding instrumentation. CubeSat technology would have to provide 3-axis-stabilized attitude control and determination with sufficient accuracy for this kind of application; <25 arcsec 3-axis has been demonstrated in Earth orbit using the Blue Canyon Technologies XACT hardware aboard the University of Colorado's Miniature X-ray Solar Spectrometer (MinXSS), which would need to be modified to operate without magnetic torquers for Mars (Mason *et al.*, 2016). CubeSats positioned in Sun-synchronous orbits with nodes spread out in local time could provide global measurements at multiple local times, suitable for data assimilation and for studying atmospheric processes that occur on timescales less than half of one martian day (see, *e.g.*, Fig. 6, right).

Another strategy is the deployment of a network of meteorological stations on the surface of Mars. The recently developed Mars_DROP_ concept enables the landing of small payloads on the surface of Mars (Staehle *et al.*, 2015). A meteorological package consisting of *in situ* sensors for pressure, temperature, and humidity, as well as potentially a deployable wind mast, would have to be sufficiently miniaturized for a Mars_DROP_ shell. A network consisting of a sufficient number of stations could provide valuable measurements for constraining local near-surface weather, as well as global weather patterns, through data assimilation. This is critical for improving our understanding of circulation and trace gas transport, and thereby relating the observed concentrations to sources and sinks on Mars.

Direct wind measurements on Mars would be desirable to better characterize martian atmospheric dynamics and enable back-trajectory simulations. Martian wind measurements require technological innovations to become a feasible scientific target. TGO will not provide substantial improvements in wind measurements.

The need for atmospheric measurement networks on Mars has long been known and articulated (Planetary Science Decadal Survey, 2011). Network sampling is necessary because martian atmospheric circulation closely interacts with the dust, CO_2_, and H_2_O cycles. Consequently, the transport, distribution, and fate of volatiles are complex and highly variable, requiring synoptic/network sampling to advance MGCM development. Although ExoMars TGO will map key trace volatiles and isotopologues with unprecedented sensitivity, it will not address the need for network measurements. Indeed, the Planetary Science Decadal Survey (2011) states the vision as follows:

“Fundamental advances in our understanding of modern climate would come from a complete determination of the three-dimensional structure of the Martian atmosphere, from the surface boundary layer to the exosphere. This determination should be performed globally, ideally by combining measurements of wind, surface pressure, and temperature from landed and orbital payloads. Surface measurements are required in order to complement these measurements and to characterize the boundary layer and monitor accurately the long-term evolution of the atmospheric mass. On a global scale, a network of at least 16 meteorological stations would be ideal, and carrying a capable meteorological payload on all future landed missions to measure surface pressure, temperature, electrical fields, and winds would provide an excellent start to developing such a network. These investigations should be complemented by the systematic monitoring of the three-dimensional fields of water vapor, clouds, and surface frosts. Isotopic signatures of volatiles (such as heavy water, HDO) should also be monitored to investigate the signature of ancient reservoirs and to study fractionation processes (*e.g.*, cloud microphysics). Finally, research and analysis should continue in order to improve the numerical climate modeling of the key atmospheric processes and to support laboratory research, notably in relation to the properties of carbon dioxide ice and its behavior under Martian conditions.”

Cost realism has prevented substantial progress toward such an assimilated measurement program, although the advent of interplanetary SmallSats can change this landscape. We envision that multiple small instruments will fit into the ballast space of larger spacecraft, permitting future Mars missions to deploy multiple assets with minimal additional cost to the primary mission. In particular, photonic-integrated-circuit (PIC) technology can enable chip-sized sensors and complete interplanetary instruments in a cell phone form factor. Such technology can enable affordable deployment of multiple sensors capable of making simultaneous measurements from different locations and/or platforms (surface, airborne, orbital), as well as flexible/adaptive deployment approaches. For example, PIC heterodyne sensors can measure trace gases, isotope ratios, and wind speeds via solar occultation. Heterodyne spectrometers offer ultrahigh spectral resolution, without bulky dispersive elements or moving parts, and shot-noise-limited signal-to-noise ratio. PIC technology enables heterodyne spectrometers-on-a-chip residing in the focal plane of a minitelescope to detect sunlight that has propagated through the planetary atmosphere. Amplitudes of absorption lines observed in the sunlight indicate molecular abundances, while ratios between different rotational/vibrational lines indicate atmospheric temperatures (Mahieux *et al.*, 2010; Vandaele *et al.*, 2015). In addition, Doppler shifts in line-center wavelengths indicate wind speeds (Sonnabend *et al.*, 2006). These nanospectrometers could conduct limb observations from orbital and airborne platforms, as well as upward-looking observations from CubeSat-sized landers.

## 9. Conclusions

The CH_4_ measurements by MSL-TLS raise two fundamental challenges. First, the observed seasonal variability of CH_4_ is incompatible with the standard models for the physics and chemistry of the martian atmosphere. Second, the observed bursts of CH_4_ demand thus far unknown sources and/or modulation mechanisms on Mars. We take a broader view of the possible origins of CH_4_ on Mars to include more general questions related to the present and past habitability of Mars and the origin of life on Mars. Existing hypotheses for martian CH_4_ sources include gas–water–rock chemistry and microbes (either extinct or extant methanogens). If proven, the former implies the existence of environments offering liquid water and chemical sources of energy—that is, habitability—while the latter implies the discovery of life on Mars.

Resolving the CH_4_ sources and sinks on Mars will require model development and technology development strategies. Solving these planetary-scale puzzles requires a concerted research effort across many disciplines. The following is a summary listing the main recommendations of this article:

(1) Advancing our understanding of the potential for life and habitability on Mars requires an interdisciplinary study of atmospheric chemistry and dynamics, subsurface biogeochemistry, astrobiology, planetary geology, and geophysics (Section 1).
(2) The martian atmosphere and surface are an overwhelmingly oxidizing environment that is inhospitable to life, since life requires redox gradients as an essential source of energy. There should be a concerted effort to search for subsurface redox gradients that could serve as energy sources for life on Mars (Sections 4 and 5).
(3) To discriminate between abiotic and biological sources of CH_4_ on Mars, future work should include studies of CH_4_ isotopes, alkane abundance ratios, H_2_ concentration, and species such as acetic acid. More insight is needed on serpentinization and FTT reactions, specifically under which conditions they effectively operate (temperature, pressure, catalysts) and how widespread such processes occur on ancient and modern-day Mars (Section 4).
(4) Seepage should exist on Mars, and it may provide natural access for characterizing the martian subsurface. Specific surface manifestations in association with faults and fractured rocks, analogous to those observed on Earth, can indicate seepage on Mars. Geological terrains characterized by regional faulting or containing apparent mud volcanoes, preferably above or near regions with evidence for alteration of mafic or ultramafic igneous rocks or sedimentary rocks by hydration, are the most promising places to search for seepage on Mars (Section 5).
(5) A hitherto unknown process is responsible for the short lifetime of CH_4_ on Mars. Powerful oxidants, such as O, O_3_, H_2_O_2_, nitrate, and perchlorate, exist in the atmosphere or on the surface, and they can possibly shorten the lifetime of methane. However, any plausible new candidate processes that destroy CH_4_ must not significantly change the well-determined lifetimes of atmospheric species such as CO and H_2_ (Section 3).
(6) TLS CH_4_ measurements on Mars have demonstrated a need for back-trajectory analyses to track down methane sources. A realistic MGCM requires assimilation of global observations (Section 8).
(7) Global observations require the deployment of new assets to measure atmospheric profiles of wind, surface pressure, temperature, dust, water/ice, and water vapor. Measurements need to have continuous global coverage to be useful for assimilation, motivating technological innovation (Section 8).
(8) New technologies suited for CubeSat/SmallSat-inspired platforms are key to enabling feasible measurement networks on Mars to perform observations of atmospheric composition, wind, surface pressure, temperature, dust, and water/ice profiles. Ideally, the network should comprise multiple vantage points (*e.g.*, orbital and land based) to provide global, 4D (spatial and temporal) characterization (Section 8).

In addition, chemical characterization of the martian subsurface (down to the theoretical groundwater stability depth, which can be as deep as a few km) is pivotal to better constrain the sources of methane. Ideally, we need (a) higher resolution and deeper subsurface sounding (*e.g.*, radar and preferably low-frequency electromagnetic sounding) to obtain a more global and refined understanding of the subsurface volatile, liquid water, brine, ice, and clathrate inventory and (b) drilling operations to characterize the chemical composition and redox state of the subsurface (Sections 4 and 8).

As we conclude the writing of this article, we anticipate initial announcements of CH_4_ measurement results from the ExoMars mission. This will be a major signpost in the pursuit of understanding methane on Mars, no matter the outcome. If ExoMars observes significant levels of methane on Mars, the global mapping with unprecedented sensitivity will guide future exploration strategies. On the contrary, a nondetection by ExoMars will not rule out localized hotspots obscured by atmospheric mixing/dilution in the large spatial resolution volume of the instruments or vertical trapping (by the atmospheric boundary layer, preventing methane from reaching observable altitudes) (Fonseca et al., 2018). Thus, either of these scenarios will, in fact, elevate the need to better understand the dynamics of methane transport/mixing on Mars and the need to implement the measurement and modeling capabilities discussed in Section 8.

Eigenbrode *et al.* (2018) and Orosei *et al.* (2018) recently reperted discoveries of complex organic compounds and ground water on Mars. These discoveries might have implications for the origin of CH_4_, although we have not had the chance to study these implications in earnest.

## Abbreviations Used

ACS: Atmospheric Chemistry Suite
CH_4_: methane
FTT: Fischer–Tropsch type
GCM: general circulation model
LMD: Laboratoire de Météorologie Dynamique
MEX: Mars Express
MGCMs: Mars general circulation models
MSL: Mars Science Laboratory
NOMAD: Nadir and Occultation for Mars Discovery
PIC: photonic-integrated-circuit
ppbv: parts per billion by volume
SAM: Sample Analysis at Mars
TGO: Trace Gas Orbiter
TLS: Tunable Laser Spectrometer

## Appendix 1

### A Synopsis of Non-TLS-MSL Mars Methane Measurements

Ground-based telescopic and Mars Express (MEX) orbital observations of CH_4_ on Mars have been extremely interesting, but remain tentative.

The first reports of CH_4_ in Mars' atmosphere, by both MEX and ground-based observations (at 10 parts per billion by volume [ppbv] level), stirred up excitement in the scientific community (Formisano *et al.*, 2004; Krasnopolsky *et al.*, 2004) and immediately raised questions regarding the origin of that CH_4_. On Mars, oxidants and UV radiation readily destroy atmospheric methane. Therefore, the presence of atmospheric CH_4_ requires ongoing or recent emission. On Earth, CH_4_ is predominantly of (live and fossil) biological origin, while abiotic sources, believed to be predominantly volcanic and/or hydrothermal (both high-temperature and low-temperature water/rock reaction hydrothermal processes), account for the rest (a few percent) of the total CH_4_ flux into the atmosphere (Etiope and Sherwood Lollar, 2013). The observations of CH_4_ in Mars' atmosphere turn the theoretical musings of possible life on Mars into a necessary investigative component for interpreting observational data. The findings elevate the possibility of hydrothermal activity on Mars, which could furnish habitable environments by providing liquid water and redox energy to sustain life. Moreover, these findings compel new research directions to explore novel processes that can produce CH_4_ on Mars and revise our understanding of the basic physical, chemical, and geological processes taking place on the planet.

Subsequent to 2004, several teams reported high spatial and temporal variability, including plumes of up to 60 ppbv CH_4_. This variability posed an even greater theoretical challenge than the mere presence of CH_4_. Methane's predicted 300-year atmospheric lifetime is far longer than the global mixing time of about 1 month (Lefèvre and Forget, 2009; Mumma *et al.*, 2009; Fonti and Marzo, 2010; Geminale *et al.*, 2011). Hence, CH_4_ should be uniformly distributed in the martian atmosphere. Introducing novel, much more efficient sinks can shorten the predicted lifetime of methane (Atreya *et al.*, 2006). However, a strong sink, whatever its nature, would also require a source that seems implausibly strong. Indeed, Lefèvre and Forget (2009) stated that the observed variations of CH_4_ on Mars are not explained by standard models of atmospheric chemistry and physics. Previous remote-sensing detection claims have been called into question due to interference from telluric absorption in the ground-based observations, low spectral resolution in the orbital observations, and contradictions between the locations of maxima reported from ground-based observations and maps obtained by the Planetary Fourier Spectrometer and Thermal Emission Spectrometer (Lefèvre and Forget, 2009; Zahnle *et al.*, 2011; Kerr, 2012).

Given the puzzling nature of observations of CH_4_ on Mars, it is highly desirable to obtain simultaneous measurements over specific locations using more than one instrument, for example, Mars Science Laboratory (MSL)-Tunable Laser Spectrometer (TLS) and ground-based observations, or MSL-TLS and Trace Gas Orbiter. We consider the confirmation of CH_4_ by two independent observations a high priority for the future exploration of Mars.
